# Supplementary material for: Molecular evidence of SARS-CoV-2 in New York before the first pandemic wave
Source: Nat Commun. 2021 Jun 8;12:3463. doi: 10.1038/s41467-021-23688-7 (PMC8187428; doi:10.1038/s41467-021-23688-7)
Supplement: Supplementary file 1 — Supplementary Information [file 41467_2021_23688_MOESM1_ESM.pdf]

Molecular evidence of SARS-CoV-2 in New York before the first pandemic wave

SUPPLEMENTARY INFORMATION

Supplementary Fig. 1. Hospital distribution and SARS-CoV-2 quantitation of negative pools. a 304 RPN pools were generated from nasopharyngeal swab specimens collected at distinct MSHS sites (A-E) from 30 December 2019 to 7 March 2020 (Hospital A, blue; B, red; C, green; D, purple; E, orange). **b** SARS-CoV-2 NAAT quantities in RPN pools by week. Cycle thresholds (Ct) of pools with detectable RNA by clinical diagnostic SARS-CoV-2 RT-PCR are depicted. If only the *ORF1ab* target or only the *E* gene target was detected in a pool, an X denotes the corresponding Ct value in green or red, respectively. If both targets were detected, the average of the Ct values of both detected targets is depicted by a blue circle for that pool.

Supplementary Fig. 1

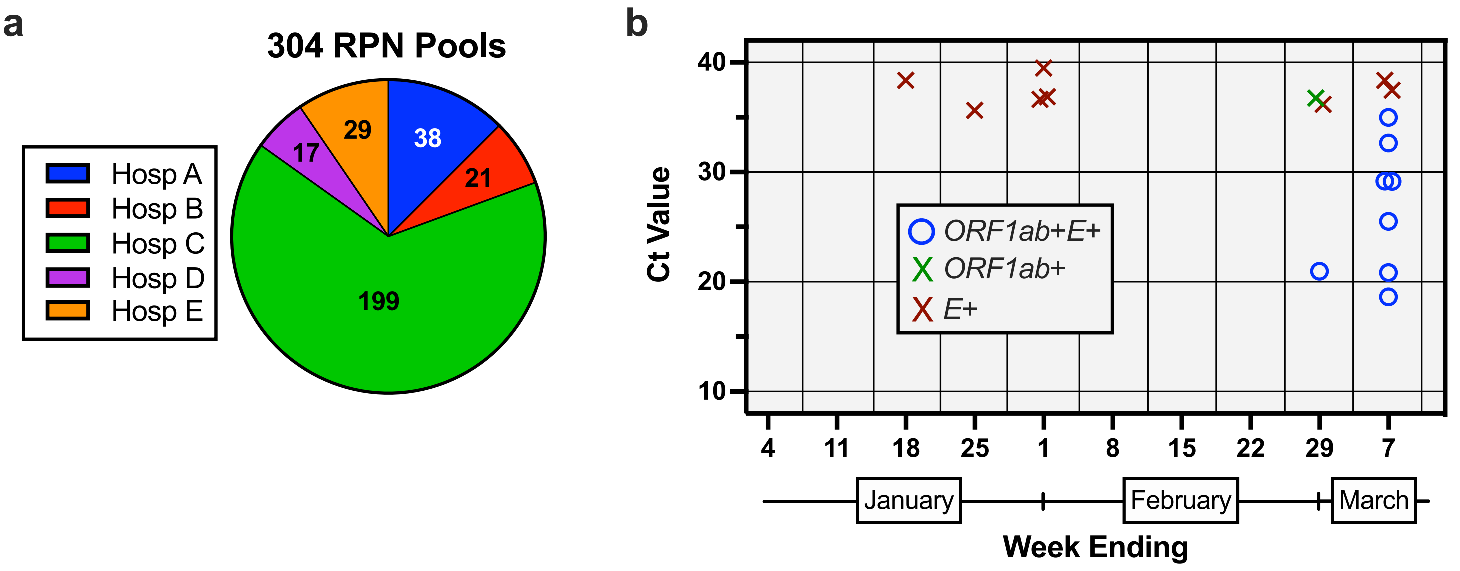

Supplementary Fig. 1. Hospital distribution and SARS-CoV-2 quantitation of negative pools. (A) 304 RPN pools were generated from nasopharyngeal swab specimens collected at distinct MSHS sites (A-E) from 30 December 2019 to 7 March 2020. (B) SARS-CoV-2 NAAT quantities in RPN pools by week. Cycle thresholds (Ct) of pools with detectable RNA by clinical diagnostic SARS-CoV-2 RT-PCR are depicted. If only the *ORF1ab* target or only the *E* gene target was detected in a pool, an X denotes the corresponding Ct value in green or red, respectively. If both targets were detected, the average of the Ct values of both detected targets is depicted by a blue circle for that pool.

Supplementary Table 1. Total and SARS-CoV-2 mapped reads per library for RNP pools with incomplete genomes

					SARS-CoV-2			
**Result**	**RPN**	**Ct Values (*ORF1ab*, *E*)** [a]	**Library** [b]	**Total reads**	**Reads**	**%**	**Q30 reads**	**Q30 %**
ORF1ab+	P34	36.75, ND	Nextera XT	7965566	17443	0%	2545	0.03%
			Swift	1160563	73798	6%	3355	0.29%
			Swift-low	696604	42871	6%	6430	0.92%
ORF1ab+*E*+	P51	34.25, 35.73	Nextera XT	738641	28298	4%	24709	3.35%
ORF1ab+*E*+	P58	31.66, 33.65	Nextera XT	1795845	537177	30%	505000	28.12%
			Swift	1024048	70196	7%	36608	3.57%
			Swift-low	1130048	77639	7%	62833	5.56%
E+	P263	ND, 36.63	Nextera XT	5187253	26720	1%	95	0.00%
			Swift	1491850	39336	3%	1905	0.13%
			Swift-low	1448665	8820	1%	121	0.01%
E+	P271	ND, 39.49	Nextera XT	14412083	77720	1%	448	0.00%
			Swift	2291892	118264	5%	865	0.04%
			Swift-low	1130161	61587	5%	380	0.03%
E+	P275	ND, 35.63	Nextera XT	5890208	44788	1%	28366	0.48%
			Swift	741823	44064	6%	333	0.04%
			Swift-low	864893	35945	4%	189	0.02%

a. If Ct value is not detected, “ND” is noted.

b. Nextera XT comprises data from both whole-genome and targeted custom amplicon sequencing library preparations.

Supplementary Fig. 2. Maximum likelihood (ML) phylogenetic inference of seven SARS-CoV-2 genome sequences from this surveillance study in a global background of 2,993 (same data as Fig. 2). Tip circles indicate the position of the respiratory pathogen-negative (RPN) pools (red) described in this report, the first reported COVID-19 case in New York City (green) from 29 February, later NYC cases from MSHS (yellow) and other institutions (dark grey), and US (blue) early isolates prior to 1 March. Tips without circles correspond to the background global isolates. The yellow box delineates the position of the clade containing the majority of NYC sequences detected during the early spread. The PANGO lineage classification of the RPN pools is indicated by the dash lines, and the NextStrain clades are shown as node labels. Scale bar represents the number of nucleotide substitutions per site. The statistical support is indicated for the branches with bootstrap support values ≥70%.

Supplementary Fig. 2

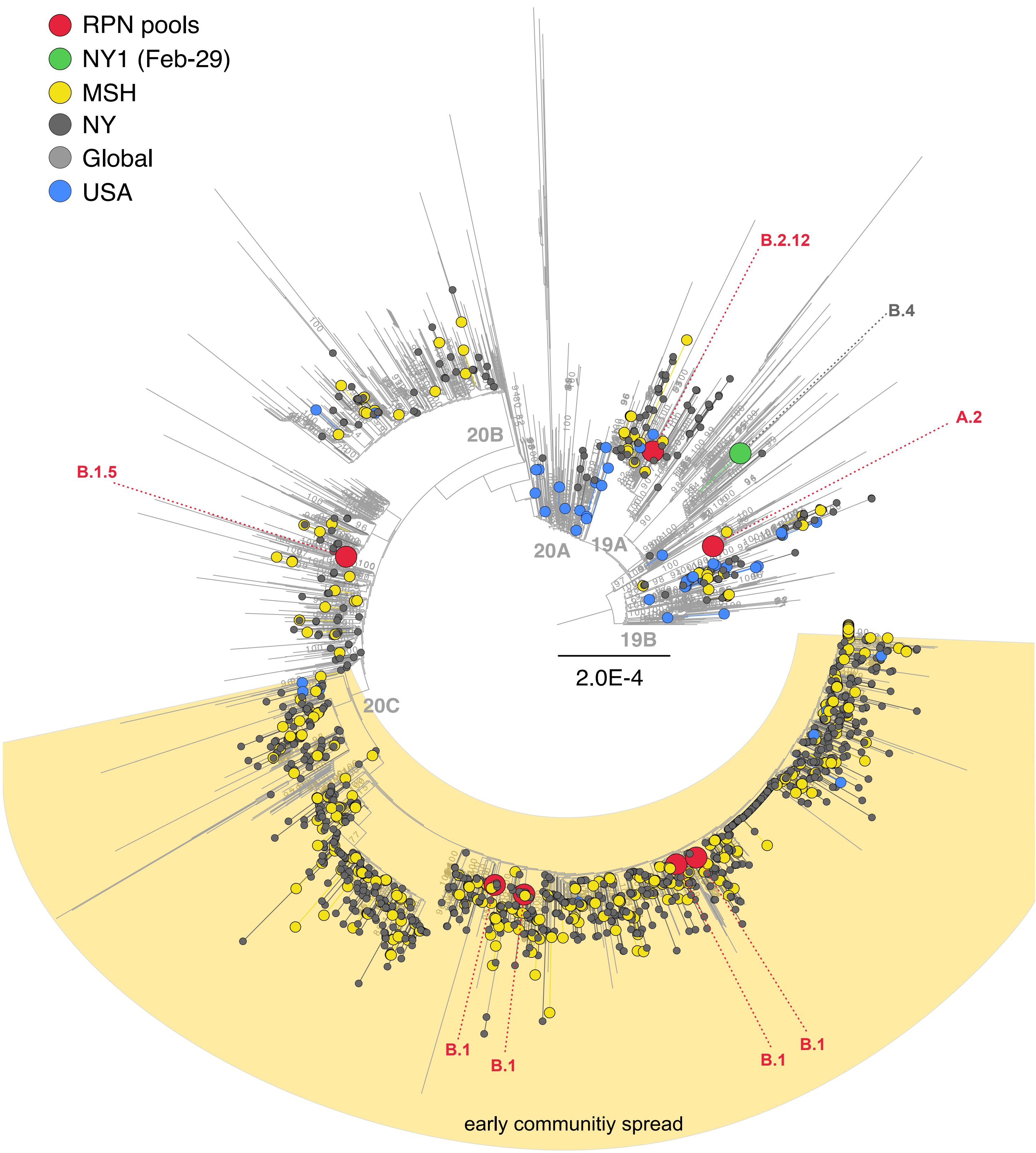

Supplementary Fig. 2. Maximum likelihood (ML) phylogenetic inference of seven SARS-CoV-2 genome sequences from this surveillance study in a global background of 2,993 (same data as Fig. 2). Tip circles indicate the position of the respiratory pathogen-negative (RPN) pools (red) described in this report, the first reported COVID-19 case in New York City (green) from 29 February, later NYC cases from MSHS (yellow) and other institutions (dark grey), and US (blue) early isolates prior to 1 March. Tips without circles correspond to the background global isolates. The yellow box delineates the position of the clade containing the majority of NYC sequences detected during the early spread. The PANGO lineage classification of the RPN pools is indicated by the dash lines, and the NextStrain clades are shown as node labels. Scale bar represents the number of nucleotide substitutions per site. The statistical support is indicated for the branches with bootstrap support values ≥70%.

Supplementary Table 2

SARS-CoV-2 primer sets for whole-genome amplification

Name (1.5 Kb set)	Sequence	Pool	Tm
covid19_1.5kb_1_LEFT	ACCAACCAACTTTCGATCTCTTGT	1	60.69
covid19_1.5kb_1_RIGHT	ATAGTGCGACCACCCTTACGAA	1	61.47
covid19_1.5kb_2_LEFT	CCACTTGCGAATTTTGTGGCAC	2	61.68
covid19_1.5kb_2_RIGHT	AGTAACCTTTGTTGGTGCACCG	2	61.58
covid19_1.5kb_3_LEFT	AGGAAGTTGTCTTGAAAACTGGTGA	1	60.81
covid19_1.5kb_3_RIGHT	CAGCGATCTTTTGTTCAACTTGCT	1	60.79
covid19_1.5kb_4_LEFT	TCAGCACGAAGTTCTACTTGCA	2	60.22
covid19_1.5kb_4_RIGHT	AGTTGGACCAAACTGTTGTCCA	2	60.29
covid19_1.5kb_5_LEFT	CCTATTCTGGACAATCTACACAACTAGG	1	60.89
covid19_1.5kb_5_RIGHT	GATTATCCATTCCCTGCGCGTC	1	61.75
covid19_1.5kb_6_LEFT	TGGCTATTGATTATAAACACTACACACCC	2	61.49
covid19_1.5kb_6_RIGHT	ACTGTAGTGACAAGTCTCTCGCA	2	61.3
covid19_1.5kb_7_LEFT	GCAATTGTTTTTCAGCTATTTTGCAGT	1	60.27
covid19_1.5kb_7_RIGHT	AGTGTACTCTATAAGTTTTGATGGTGTGT	1	60.73
covid19_1.5kb_8_LEFT	ACAAGGCTATTGATGGTGGTGTC	2	60.69
covid19_1.5kb_8_RIGHT	AGCATGTCTTCAGAGGTGCAGA	2	61.68
covid19_1.5kb_9_LEFT	GTTCTTTACCAACCACCACAAACC	1	60.68
covid19_1.5kb_9_RIGHT	GTCCACACTCTCCTAGCACCAT	1	61.48
covid19_1.5kb_10_LEFT	ACCTTCTCTTGCCACTGTAGCT	2	61.35
covid19_1.5kb_10_RIGHT	TCTGTCGTAGTGCAACAGGACT	2	61.33
covid19_1.5kb_11_LEFT	TGGTACAACATTTACTTATGCATCAGC	1	60.42
covid19_1.5kb_11_RIGHT	AATACCAGCATTTCGCATGGCA	1	61.53
covid19_1.5kb_12_LEFT	GGCATTTTGATGAAGGTAATTGTGACA	2	61
covid19_1.5kb_12_RIGHT	AACATGTTGTGCCAACCACCAT	2	61.55
covid19_1.5kb_13_LEFT	ACGTAATGTCATCCCTACTATAACTCAAA	1	60.18
covid19_1.5kb_13_RIGHT	ACAACCTGGAGCATTGCAAACA	1	61.54
covid19_1.5kb_14_LEFT	TGAGGCTATGTACACACCGCAT	2	61.8
covid19_1.5kb_14_RIGHT	CCGAGGAACATGTCTGGACCTA	2	61.21
covid19_1.5kb_15_LEFT	TCTTTGATGAAATTTCAATGGCCACA	1	60.46
covid19_1.5kb_15_RIGHT	TGTCTGAAGCAGTGGAAAAGCA	1	60.68
covid19_1.5kb_16_LEFT	GGACTTCCTTGGAATGTAGTGCG	2	61.48
covid19_1.5kb_16_RIGHT	CAGTGAGTGGTGCACAAATCGT	2	61.63
covid19_1.5kb_17_LEFT	AATGTAGCATTTGAGCTTTGGGC	1	60.37
covid19_1.5kb_17_RIGHT	TAACAAAGGCTGTCCACCATGC	1	61.4
covid19_1.5kb_18_LEFT	GGATCTCATTATTAGTGATATGTACGACCC	2	61.07
covid19_1.5kb_18_RIGHT	GGTCAAGTGCACAGTCTACAGC	2	61.44
covid19_1.5kb_19_LEFT	TGGTAGATTTGCCAATAGGTATTAACATCA	1	61.04
covid19_1.5kb_19_RIGHT	TGACTAGCTACACTACGTGCCC	1	61.52
covid19_1.5kb_20_LEFT	AGTCCCTGTTGCTATTCATGCA	2	60.03

covid19_1.5kb_20_RIGHT	CATGACAAATGGCAGGAGCAGT	2	61.46
covid19_1.5kb_21_LEFT	AGCTGCAGAAATCAGAGCTTCT	1	60.03
covid19_1.5kb_21_RIGHT	ACATGTTCAACACCAGTGTCTGT	1	60.62
covid19_1.5kb_22_LEFT	TGCTGGCATACTAATTGTTACGACT	2	60.63
covid19_1.5kb_22_RIGHT	TCAATCTCCATTGGTTGCTCTTCA	2	60.51
covid19_1.5kb_23_LEFT	TGCTTTGCTTGTACAGTAAGTGACA	1	61.14
covid19_1.5kb_23_RIGHT	TAGCACCATAGGGAAGTCCAGC	1	61.55
covid19_1.5kb_24_LEFT	GGCCCCAAGGTTTACCCAATAA	2	60.56
covid19_1.5kb_24_RIGHT	TAGGCAGCTCTCCCTAGCATTG	2	61.61
**Name (2.0 Kb set)**	**Sequence**	**Pool**	**Tm**
covid19_2kb_1_LEFT	ACCAACCAACTTTCGATCTCTTGT	1	60.69
covid19_2kb_1_RIGHT	ACACCACCTGTAATGTAGGCCA	1	61.36
covid19_2kb_2_LEFT	GGTGCCTGGAATATTGGTGAACA	2	61.13
covid19_2kb_2_RIGHT	GCCGACAACATGAAGACAGTGT	2	61.38
covid19_2kb_3_LEFT	TGGAACTTACACCAGTTGTTCAGAC	1	61.14
covid19_2kb_3_RIGHT	AGCATCTTGTAGAGCAGGTGGA	1	61.16
covid19_2kb_4_LEFT	AACCTCATAATTCACATGAAGGTAAAACA	2	60.03
covid19_2kb_4_RIGHT	AAAACACNTAAAGCAGCGGTTGA	2	61.49
covid19_2kb_5_LEFT	AAACCGTGTTTGTACTAATTATATGCCTT	1	60.13
covid19_2kb_5_RIGHT	TCACGAGTGACACCACCATCAA	1	61.86
covid19_2kb_6_LEFT	ACGAAAACAAATACGTAGTGCTGC	2	60.61
covid19_2kb_6_RIGHT	TGGGCCTCATAGCACATTGGTA	2	61.5
covid19_2kb_7_LEFT	TCTGAAGACATGCTTAACCCTAATTATGA	1	60.59
covid19_2kb_7_RIGHT	ATCACCATTAGCAACAGCCTGC	1	61.53
covid19_2kb_8_LEFT	ATTGTGGGCTCAATGTGTCCAG	2	61.14
covid19_2kb_8_RIGHT	AGCATAGACGAGGTCTGCCATT	2	61.54
covid19_2kb_9_LEFT	GCTAAATTCCTAAAAACTAATTGTTGTCGC	1	60.82
covid19_2kb_9_RIGHT	TGCATTAACATTGGCCGTGACA	1	61.4
covid19_2kb_10_LEFT	CCTAAATGTGATAGAGCCATGCCT	2	60.51
covid19_2kb_10_RIGHT	TGCGAGCAGAAGGGTAGTAGAG	2	61.27
covid19_2kb_11_LEFT	TGTTGGTGATTATTTTGTGCTGACAT	1	60.24
covid19_2kb_11_RIGHT	CAAAGCACTCGTGGACAGCTAG	1	61.49
covid19_2kb_12_LEFT	CTGAGCGCACCTGTTGTCTATG	2	61.56
covid19_2kb_12_RIGHT	TGAACCTGTTTGCGCATCTGTT	2	61.59
covid19_2kb_13_LEFT	ACCCAGGAGTCAAATGGAAATTGA	1	60.45
covid19_2kb_13_RIGHT	CCTGAGGGAGATCACGCACTAA	1	61.53
covid19_2kb_14_LEFT	CAATTTTGTAATGATCCATTTTTGGGTGT	2	60.29
covid19_2kb_14_RIGHT	TGCTGCATTCAGTTGAATCACCA	2	61.31
covid19_2kb_15_LEFT	ACCCATTGGTGCAGGTATATGC	1	60.75
covid19_2kb_15_RIGHT	TGCAGTAGCGCGAACAAAATCT	1	61.44
covid19_2kb_16_LEFT	CCATAGTAATGGTGACAATTATGCTTTGC	2	61.57
covid19_2kb_16_RIGHT	AGCGTTCGTGATGTAGCAACAG	2	61.49
covid19_2kb_17_LEFT	TGTGGCTCAGCTACTTCATTGC	1	61.2

covid19_2kb_17_RIGHT	TAGCACCATAGGGAAGTCCAGC	1	61.55
covid19_2kb_18_LEFT	ACTTGTCACGCCTAAACGAACA	2	60.74
covid19_2kb_18_RIGHT	TAGGCAGCTCTCCCTAGCATTG	2	61.61
