# Supplementary material for: Molecular evidence of SARS-CoV-2 in New York before the first pandemic wave
Source: Nat Commun. 2021 Jun 8;12:3463. doi: 10.1038/s41467-021-23688-7 (PMC8187428; doi:10.1038/s41467-021-23688-7)
Supplement: Supplementary file 3 — Reporting Summary [file 41467_2021_23688_MOESM3_ESM.pdf]

nature research

Corresponding author(s): Sordillo, Emilia Mia

Last updated by author(s): Apr 26, 2021

# Reporting Summary

Nature Research wishes to improve the reproducibility of the work that we publish. This form provides structure for consistency and transparency in reporting. For further information on Nature Research policies, see our Editorial Policies and the Editorial Policy Checklist.

## Statistics

For all statistical analyses, confirm that the following items are present in the figure legend, table legend, main text, or Methods section.

| n/a | Confirmed | |
|---|---|---|
| ☒ | ☐ | The exact sample size (*n*) for each experimental group/condition, given as a discrete number and unit of measurement |
| ☒ | ☐ | A statement on whether measurements were taken from distinct samples or whether the same sample was measured repeatedly |
| ☒ | ☐ | The statistical test(s) used AND whether they are one- or two-sided<br>*Only common tests should be described solely by name; describe more complex techniques in the Methods section.* |
| ☒ | ☐ | A description of all covariates tested |
| ☒ | ☐ | A description of any assumptions or corrections, such as tests of normality and adjustment for multiple comparisons |
| ☒ | ☐ | A full description of the statistical parameters including central tendency (e.g. means) or other basic estimates (e.g. regression coefficient) AND variation (e.g. standard deviation) or associated estimates of uncertainty (e.g. confidence intervals) |
| ☒ | ☐ | For null hypothesis testing, the test statistic (e.g. *F*, *t*, *r*) with confidence intervals, effect sizes, degrees of freedom and *P* value noted<br>*Give P values as exact values whenever suitable.* |
| ☒ | ☐ | For Bayesian analysis, information on the choice of priors and Markov chain Monte Carlo settings |
| ☒ | ☐ | For hierarchical and complex designs, identification of the appropriate level for tests and full reporting of outcomes |
| ☒ | ☐ | Estimates of effect sizes (e.g. Cohen's *d*, Pearson's *r*), indicating how they were calculated |

*Our web collection on statistics for biologists contains articles on many of the points above.*

## Software and code

Policy information about availability of computer code

| | |
|---|---|
| Data collection | No software was used for data collection. |
| Data analysis | Illumina data were analyzed using a custom reference-based (MN908947.3) pipeline, https://github.com/mjsull/COVID_pipe, to reconstruct SARS-CoV-2 genomes. Phylogenetic relationships of the 7 high-quality consensus sequences (>80% completeness) were inferred over a global background of SARS-CoV-2 sequences between December 2019 and April 2020 downloaded from Global Initiative on Sharing Avian Influenza Data (GISAID). Initial alignment and subsampling were done by using the NextStrain tool (v1.0, build February 17, 2021). A maximum likelihood (ML) phylogeny was inferred with IQ-TREE under the GTR+F+I+G4 model, after which further manual curation was done to identify and remove extreme outliers that deviated from a temporal signal using Tempest. The final ML tree containing 3,700 taxa was then time-scaled with TreeTime using a skyline coalescent tree prior and a strict clock model. Lineage classification was done using a phylogenetic based nomenclature as described by Rambaut et al, 2020. using the PANGOLIN tool, lineages version 2020-10-03 (O'Toole & McCrone, 2020). |

For manuscripts utilizing custom algorithms or software that are central to the research but not yet described in published literature, software must be made available to editors and reviewers. We strongly encourage code deposition in a community repository (e.g. GitHub). See the Nature Research guidelines for submitting code & software for further information.

## Data

Policy information about availability of data

All manuscripts must include a data availability statement. This statement should provide the following information, where applicable:

- Accession codes, unique identifiers, or web links for publicly available datasets
- A list of figures that have associated raw data
- A description of any restrictions on data availability

SARS-CoV-2 sequencing read data for all study isolates and sample pools were deposited in SRA. BioProjectID PRJNA717974 [https://www.ncbi.nlm.nih.gov/sra/

PRJNA717974], BioSample accesions SAMN18520300 [https://www.ncbi.nlm.nih.gov/biosample/18520300] to SAMN18520311 [https://www.ncbi.nlm.nih.gov/biosample/18520311].

# Field-specific reporting

Please select the one below that is the best fit for your research. If you are not sure, read the appropriate sections before making your selection.

☒ Life sciences ☐ Behavioural & social sciences ☐ Ecological, evolutionary & environmental sciences

For a reference copy of the document with all sections, see nature.com/documents/nr-reporting-summary-flat.pdf

# Life sciences study design

All studies must disclose on these points even when the disclosure is negative.

| | |
|---|---|
| Sample size | All residual respiratory- pathogen negative nasopharyngeal specimens remaining after routine testing were included without limitation |
| Data exclusions | No data were excluded |
| Replication | Whole genome sequencing served as the confirmatory assay for the direct nucleic acid amplification assays. |
| Randomization | This was not a clinical trial or comparison. Respiratory pathogen negative pools were prepared by mixing aliquots from residual nasopharyngeal specimens in viral transport medium that previously tested negative during routine clinical evaluation for respiratory pathogens using multiplex diagnostic panels. Specimens had been collected at MSHS hospitals and outpatient facilities between 30 December 2019 and 7 March 2020 from patients with respiratory symptoms. These specimens had not previously been tested for SARS-CoV-2. |
| Blinding | Residual specimens were deidentified and pooled before testing. |

# Reporting for specific materials, systems and methods

We require information from authors about some types of materials, experimental systems and methods used in many studies. Here, indicate whether each material, system or method listed is relevant to your study. If you are not sure if a list item applies to your research, read the appropriate section before selecting a response.

## Materials & experimental systems

| n/a | Involved in the study |
|---|---|
| ☒ | ☐ Antibodies |
| ☒ | ☐ Eukaryotic cell lines |
| ☒ | ☐ Palaeontology and archaeology |
| ☒ | ☐ Animals and other organisms |
| ☒ | ☐ Human research participants |
| ☒ | ☐ Clinical data |
| ☒ | ☐ Dual use research of concern |

## Methods

| n/a | Involved in the study |
|---|---|
| ☒ | ☐ ChIP-seq |
| ☒ | ☐ Flow cytometry |
| ☒ | ☐ MRI-based neuroimaging |
